# Acorn Ants May Create and Use Two Entrances to the Nest Cavity

**DOI:** 10.3390/insects12100912

**Published:** 2021-10-06

**Authors:** Sławomir Mitrus

**Affiliations:** Institute of Biology, University of Opole, Oleska 22, 45-052 Opole, Poland; smitrus@uni.opole.pl

**Keywords:** *Temnothorax crassispinus*, nest site, entrance modification, multiple entrances, nest cavity, cavity-nesting ant

## Abstract

**Simple Summary:**

Large nests of ants, or ant hills, which are inhabited by numerous workers, are universally known. However, many ant species live in small colonies and do not construct nests, but instead dwell in ready-for-use cavities. In Central Europe, acorn ants are among the most widely distributed and common species but are also frequently overlooked as the workers are small and colonies of the species typically range from only a few dozen to about two hundred individuals. Often, a whole colony lives in just one empty acorn or a cavity inside a small twig. Such a cavity, such as an empty acorn, typically has one hole resulting from the activity of wood-boring beetles, and that hole is used by the ant colony as the entrance to the nest. Acorn ants have been the subject of numerous experiments, including those focused on the choice of nest sites. For example, it was previously found that ants prefer sites with a narrow vs. a wider entrance. However, cavities with good-sized hole are rare; thus, the possibility to modify a potential nest site, including a reduction in the size of the hole, should be a favorable matter for the ants. The results of this study showed that the ant colonies could inhabit imperfect cavities that need a modification, e.g., a reduction of the available holes, and that such small colonies may even create two entrances to the nest cavity. However, the effect of the presence of more than one entrance to the nest on the behavior of the ants is unknown.

**Abstract:**

Many ant species construct large nests that are inhabited by numerous workers, but other species dwell in ready-for-use cavities and live in small colonies. Ants of the genus *Temnothorax* inhabit small cavities, e.g., in acorns, twigs, and under rocks. Although a preference for nest sites with a narrower entrance is known, recent studies have shown that they also use cavities with wider entrances and may modify the size of such entrances. As good cavities for nest sites are a limited resource, the possibility to modify a potential nest site, including a reduction in the size of the hole, should be a favorable matter for the ants. Through field and laboratory experiments, I studied the acorn ant *Temnothorax crassispinus*. Observations showed that they readily inhabited imperfect cavities and, if necessary, modified the holes to such cavities. If they had to repair a nest site, they sometimes created a second entrance; there was no difference in the sizes of the entrances. In the field, for entrance modification or blocking an unnecessary hole, the acorn ants used soil, grains of sand, and parts of plants. In the laboratory, the ant colonies showed no preference for nest sites with one entrance vs. a nest cavity with two entrances. The results of this study showed that even such small ant colonies could use nest sites with multiple entrances; however, the effect of the presence of more than one entrance on the behavior of the ants is unknown.

## 1. Introduction

Most social insects, including ants, use nest sites. These nests provide a refuge against predators, ensure optimal conditions for the brood development, and can protect ants from variable conditions [1,2]. While several ant species create and use large nests inhabited by numerous workers, others inhabit ready-for-use cavities, e.g., spaces in wood or empty seeds, where they live in small colonies [1,2,3]. Such ant species, in what is sometimes called ‘cavity nesting’, frequently inhabit cavities with holes of certain sizes [4], and the diversity of the holes can affect the structure of the ant communities (e.g., [5,6]). Thus, the size of hole that can be used as an entrance to a potential nest cavity is an important factor influencing the ants.

The nest entrance, as well as the number of entrances, can facilitate protection against predators and pathogens [2,7], but is also important for the social interface [7]. For example, a high rate of contact between foragers and inner nest workers occurs near the nest entrance, which has an influence on the behavior of the colony [8,9,10]. However, good nesting sites are limited resources [1,11,12,13,14,15]. Thus, for cavity nesting ants, finding a cavity of a certain volume and with a certain-sized hole can be difficult. Therefore, the possibility to modify the nest site, including modifying the size of the hole, could be an important matter; for example, it has been shown that modification of the entrance is a widespread practice in arboreal ant species that dwell in tree cavities [16].

Examples of ants that live in small colonies are the species of the genus *Temnothorax*, which is one of the most species-rich ant genera in the world [17]. The ants of the genus typically inhabit ready-for-use cavities, such as empty acorns and hazelnuts, but also dwell in twigs, galls, rocks, or under bark [2,17,18,19]. These ants have been studied in numerous experiments covering multiple aspects, including their choice of nest sites (e.g., [20,21,22,23,24,25,26,27,28]). It was found that the ants select a nest from the various sites available; for example, they prefer dark vs. bright nest sites, thick vs. thin sites, and sites with narrow vs. wider entrances. The ants are able to compare the different nest sites and choose the better one, even if it is much further away than a worse one [23].

The natural cavities used by acorn ants from the genus *Temnothorax* typically have one hole used as the entrance to the nest. Usually, the ants inhabit cavities in empty acorns and twigs, and such cavities have just one hole resulting from the activity of a wood-boring beetle [4,29]. Nonetheless, it has been shown that the acorn ants can modify the entrance to the nest cavity [20,30]. Under natural conditions, good nest sites are limited resources for the ants [11,13,14,15]; many potential nest sites could need entrance modification. The aim of the study was check if the ants inhabit nest sites that need additional work (reducing holes) to the same proportion as sites that do not need such work, and how they repair damaged nest sites. Additionally, I performed a laboratory experiment to check if the ant colonies show a preference for nest sites with one entrance vs. two entrances.

## 2. Materials and Methods

### 2.1. The Study Species and the Study Site

I performed two field experiments and one laboratory experiment using colonies of the acorn ant *Temnothorax crassispinus*. This ant lives in light coniferous and mixed forests, is present throughout Central and Eastern Europe, and is widely distributed in Poland [2,18]. Colonies of the ant are small, typically ranging from a few dozen to about 200 workers. The workers are only about 2–4 mm in length [2,18]. The ants dwell mostly in empty acorns and small twigs [2,18,31], where the larvae of other insects have earlier bored cavities [29,32].

Both the field experiments (see below) were conducted in a beech-pine forest with a few oaks (Opole District, Poland, GPS: 50°37′29″ N, 18°06′32″ E). The presence of numerous *T. crassispinus* ant colonies was confirmed in the area prior to the start of the experiments. The ant colonies used during the laboratory experiment were also collected from Opole District (GPS: 50°34′53″ N, 18°10′57″ E).

### 2.2. Experiment 1 (Field Experiment): Do Acorn Ants Inhabit and Modify Imperfect Nest Sites?

**Figure 1:**
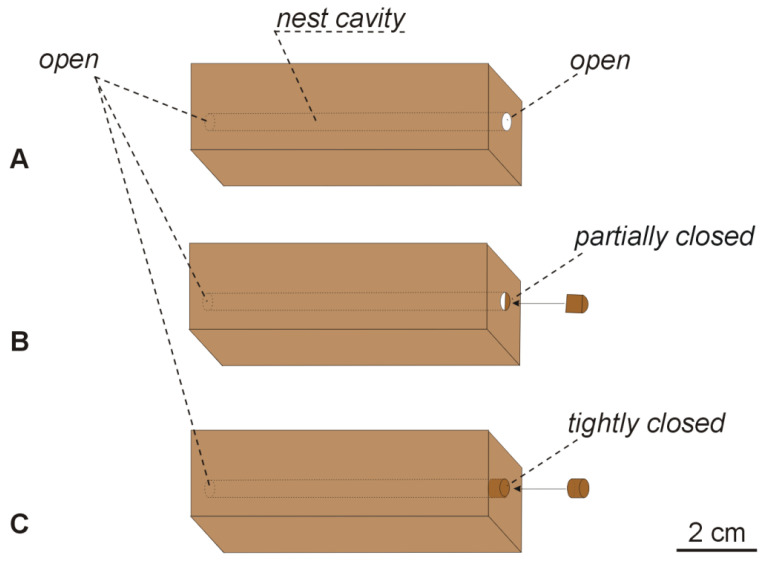
Nest designs of the artificial nest sites used during the field experiments. Three types of nest sites were used: (**A**) ‘open’—woodblock drilled lengthwise to form a 4 mm hole, where the hole was not closed; (**B**) ‘partially closed’—a hole was partially closed on one side with a beech plug; (**C**) ‘with one side closed’—a hole was fully closed on one side with a beech plug. The cavity volume was approximately 0.9 cm^3^.

Although during laboratory experiments it was shown that ant colonies prefer cavities with narrower entrances (e.g., [33]), in a previous field experiment [30], it was demonstrated that the ant colonies inhabited artificial nest sites with narrower and wider entrances in a similar proportion. The experiment was focused on checking if the ant colonies will inhabit good cavities but without good entrances. For this purpose, similarly to the previous study [30], I used a beech woodblock measuring 7.5 cm × 2.0 cm × 2.0 cm, drilled lengthwise to form a 4-mm hole and closed on one side with a beech plug. The final volume of the cavity was approximately 0.9 cm^3^. However, in this experiment, two additional types of nest sites made of woodblocks were used, in which the holes were not closed or were only partially closed. Thus, three types (see Figure 1) of potential nest sites were created:

(1) ‘Open’—woodblock drilled lengthwise, where the hole was not closed;
(2) ‘Partially closed’—woodblock drilled lengthwise, where the hole on one side was partially (halfway) closed with a beech plug, while the hole on the opposite side was not closed;
(3) ‘With one side closed’—woodblock drilled lengthwise, where the hole was closed on one side with a beech plug, while the other hole was not closed.

**Figure 2:**
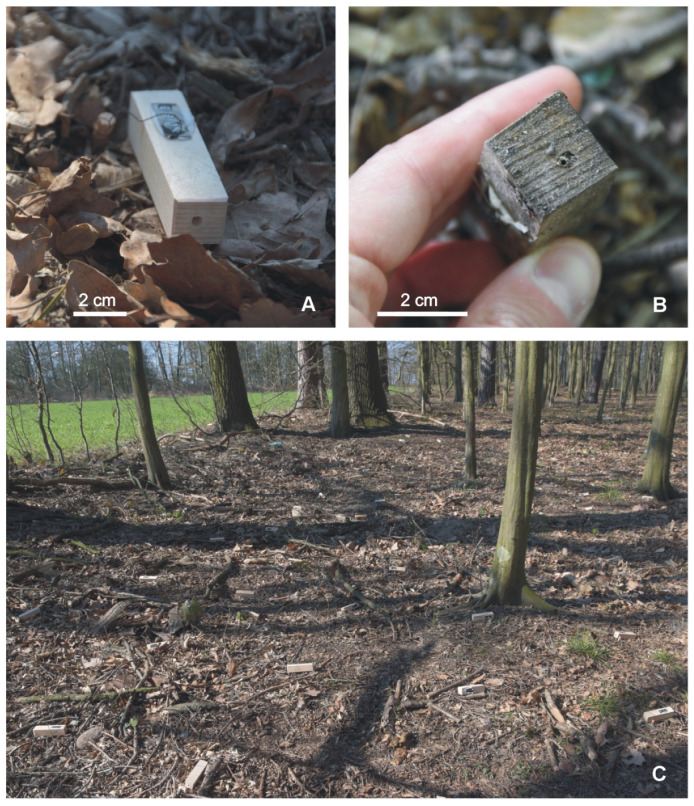
Artificial nest sites used during the field experiments. (**A**) Photograph of an artificial nest site made of a woodblock (see Figure 1). Such nest sites made of woodblocks are readily accepted by acorn ants of the genus *Temnothorax*. (**B**) Entrance to an artificial nest site modified by the *Temnothorax crassispinus* ant colony using sand and soil. (**C**) Artificial nest sites made of woodblocks during the field experiment. During this study, the artificial nest sites were located in a beech-pine forest with a few oaks, about 2–6.5 m from the edge of the forest.

I used 150 such sites, with 50 available for each of the three groups. On 25 March 2020, the woodblocks were placed in the field attached to ca. 14 cm sticks that were poked into the ground (see Figure 2A). The distance between neighboring woodblocks was ca. 50 cm and they were located about 2–6.5 m from the edge of the forest (Figure 2C). The positions of the three types of nest sites were randomized.

After about two months, on 27 May 2020, the woodblocks were collected, carefully examined, and information about modifications to the holes in the woodblocks was noted (i.e., whether the hole was completely closed with sand and soil or had been partially closed with a small round hole; left, see Figure 2B). In the forest, on the nest sites with such modifications, workers of the ant colonies were sometimes observed. Then, each woodblock was placed into a separate plastic bag and was transported to the laboratory. In the laboratory, they were again carefully examined. Where possible, the sizes of the modified entrances were measured to the nearest 0.1 mm: the diameter was measured if the entrance was round or the longest diameter of the hole and the orthogonal diameter were measured if the entrance was elliptical in shape [20]. Then, the nest sites were carefully opened and the ants captured with an aspirator and counted. The material used by the ants for the entrance modifications was collected and later observed under a stereo microscope. 

In the experiment, I wanted to check the potential for nest cavities without a good entrance hole to be accepted by the ant colonies. Since in natural conditions good nesting sites for cavity nesting ants are limited resources (see the Introduction), I predicted that the ant colonies would use and change the cavities from all the groups, but the nest sites ‘with one side closed’—as they did not require additional work—would be inhabited in a larger proportion. 

### 2.3. Experiment 2 (Field Experiment): During Nest Repairs, Do Ant Colonies Create Additional Entrances?

As I had observed that the *T. crassispinus* ant colonies may create two nest entrances to a nest cavity (see the results of Experiment 1), I performed a further experiment to check if the ant colonies might create an additional entrance when the cavity wall was partially destroyed, or if they would just block that part of the nest cavity. For this experiment, nest sites ‘with one side closed’ were used, such as those in Experiment 1 (see above and Figure 1C), i.e., a woodblock (7.5 cm × 2.0 cm × 2.0 cm), drilled lengthwise and closed on one side. During the experiment, 50 such woodblock nest sites were used. On 3 March 2021, the artificial nest sites were placed in the field. The nest sites were attached to ca. 14 cm sticks that were poked into the ground, while the distance between the nests was ca. 50 cm. They were located about 2–6.5 m from the edge of the forest (Figure 2).

During the experiment, two of the woodblocks were lost and were probably taken by animals. On 17 May, 39 of the remaining 48 nest sites had evidence of ant colonies—i.e., the hole was modified with sand and soil (see Figure 2B)—and on several of the nest sites, the workers of *T. crassispinus* were observed. Then, the beech plug closing the drilled hole was gently removed, and typically worried workers of the ant colony were observed near the opened hole. 

After another two weeks, on 31 March 2021, the woodblocks were carefully examined in the field, then collected, each placed into a separate bag, and transported to the laboratory. The procedure in the laboratory was the same as for Experiment No. 1. The opened hole could be completely blocked by ants, or the size of the hole significantly reduced using different material (typical area of such a reduced hole is about 1–1.5 mm^2^ [30], and area of initial hole of such an artificial cavity is about 12.5 mm^2^—Figure 1 and Figure 2A,B). When the hole was not completely blocked, but significantly reduced, and the initial entrance was not changed, I assumed the nest site had two entrances; additionally, in several cases, I observed, that workers used both holes as entrances to the nest cavity. I predicted that the opened hole (i.e., the destroyed part of the nest site) would be repaired—namely, it would be blocked with soil.

### 2.4. Experiment 3 (Laboratory Experiment): Do the Ant Colonies Prefer a Nest Sites with One Entrance vs. a Nest Site with Two Entrances?

**Figure 3:**
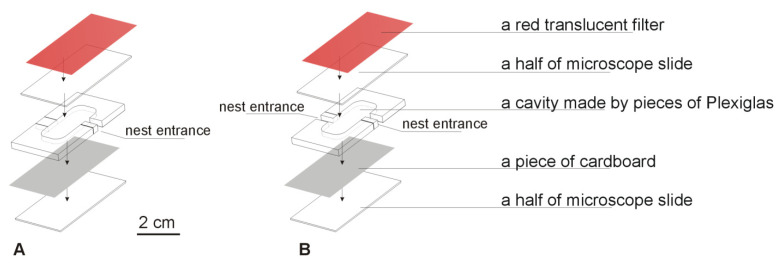
Nest design of the artificial nest sites used during the laboratory experiment: a cavity is created between a piece of cardboard and a 1/2 microscope slide (38 mm × 26 mm), which is kept apart by several pieces of Plexiglas as a frame (3 mm-thick) and coated with a piece of a red translucent filter. The volume of the cavity is ca. 0.78 cm^3^. Two types of nest sites were used: (**A**) with one entrance; and (**B**) with two entrances.

**Figure 4:**
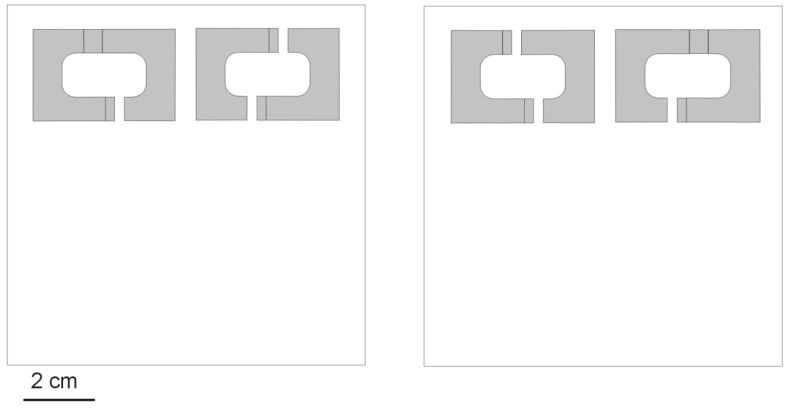
Scheme of the laboratory experiment. In square Petri dishes (10.2 cm × 10.2 cm × 1.9 cm), two artificial nest sites were placed: one with one entrance and one with two entrances (see Figure 3). The position, i.e., left or right, of the nest sites with one or two entrances was systematically varied in the Petri dishes. The ants were then released at a similar distance from the entrances to the two artificial nest sites, approximately 7 cm from the entrances.

On 31 March 2021, I collected 49 acorns containing ant colonies (6 queenless, 42 containing one queen, and one with two queens; workers ranging in number from 4–202, median: 71, mean: 75.6; and all the colonies containing brood at different stages of development). For the experiment, I chose 34 colonies containing one queen (workers: 18–202, median: 97, mean: 95.0) and transferred to square Petri dishes (10.2 cm × 10.2 cm × 1.9 cm) with a thin plaster base. Each dish contained two artificial nests comprising a cavity between a piece of cardboard and half of a microscope slide (i.e., 38 mm × 26 mm), which were kept apart by pieces of Plexiglas (3 mm-thick) and coated with a piece of a red translucent filter from above (Figure 3). The volume of the cavity was ca. 0.78 cm^3^ and the entrance size was 2.5 mm × 3 mm × 6.5 mm (width × height × length). Two types of nest sites were used: with one entrance and with two entrances, where each Petri dish contained a nest site with one entrance and a nest site with two entrances. The distance between the entrances to the two nest sites was about 7 cm (see Figure 4). The position, i.e., left or right, of the nest cavities with one or two entrances in the Petri dishes was systematically varied to eliminate any chance of a directional bias (e.g., [34]). 

The ant colonies were randomly assigned to the Petri dishes, with each ant colony in a separate dish. They were placed at a similar distance (approximately 7 cm) from the entrances to the two artificial nest cavities (see Figure 4). After 48 h, and again after six days, it was noted which nest cavity was inhabited by the ant colony. Next, dry sand was added about 4 cm from one of the nest cavity entrances (1 mL of sand with grains 0.6–0.8 mm in size; and 0.2 mL of grains less than 0.4 mm in size, i.e., grains that passed through a 0.8-mm sieve but not through a 0.6-mm, and grains that passed through a 0.4-mm sieve, respectively). The ants used the grains of sand for nest modifications [30], and typically used both smaller and larger grains—as such, a mixture of big and small grains increased the stability of the sand piles [35]. After the next two weeks, it was observed if the workers from the colonies inhabiting the nest sites with two entrances were using one or both entrances.

During the experiment, the ants were fed with frozen Dubia roach (*Blaptica dubia*; length: approximately 12 mm) and honey twice a week, while water was provided ad libitum. The Petri dishes with the ant colonies were kept in a thermostatic cabinet. A daily cycle of 12 h (12 h/light, 20 °C; dark, 10 °C) was used to mimic artificial spring conditions [36]. Despite the fact that, during laboratory experiments using *Temnothorax albipennis* ant colonies, it was shown the ants they did not distinguish between nest sites with one entrance and sites with two entrances [22], I predicted that the ant colonies would prefer the cavity with one entrance, particularly as the nest sites used during this experiment had quite large entrances.

### 2.5. Statistical Analyses

I used the chi-square test to check if the three types of cavities were inhabited by the ant colonies in similar proportions (also taking into consideration information about the presence of a queen in the colony in Experiment 1, the field experiment). For the data obtained in the experiment, I used the two-way analysis of variance to check if the colony size (i.e., number of workers) was similar in the colonies collected from the three types of nests, and to compare the sizes of the queenright vs. queenless colonies. For the queenright colonies inhabiting nest sites that were ‘partially closed’ or ‘with one side closed’, I used the two-way analysis of variance to check if the colony size was dependent on the type of nest and number of entrances. 

I used the paired-sample Student *t* test to compare the sizes of both entrances—the initial entrance, and the entrance created after the beech plug was removed, damaging the wall of the nest (Experiment 2, the field experiment). I also used the chi-square test to check if the nest sites with one entrance vs. the nest sites with two entrances were inhabited by the ant colonies in similar proportions (in Experiment 3, the laboratory experiment). 

All abovementioned statistical analyses were carried using Statistica, Ver. 13.3 software package [37]. I checked the assumptions for normality of the distribution and the homogeneity of variances prior to the analyses. The threshold for significance was *p* = 0.05 throughout. All the probability values shown are two-tailed.

## 3. Results

### 3.1. Experiment 1. (Field Experiment): Do Acorn Ants Inhabit and Modify Imperfect Nest Sites?

**Figure 5:**
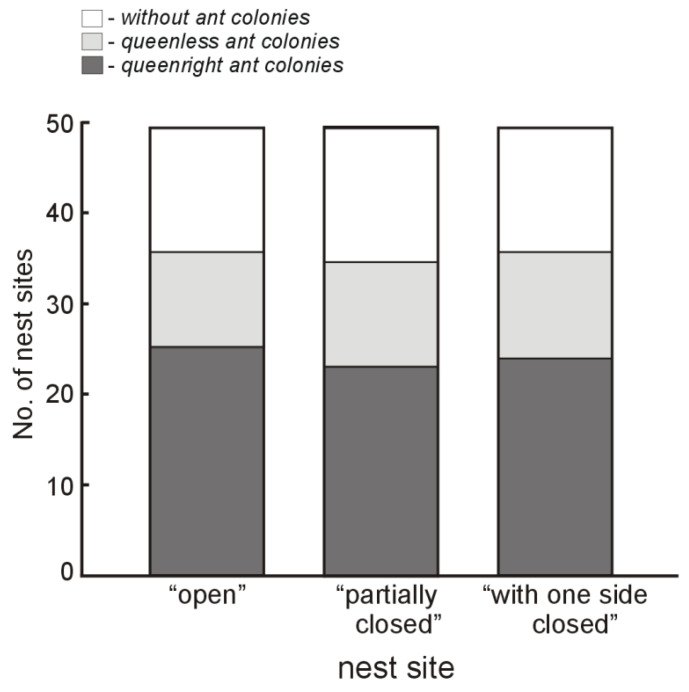
Number of artificial ‘open’, ‘partially closed’, and ‘with one side closed’ (see Figure 1) nest sites in which *Temnothorax crassispinus* ant colonies (queenright and queenless, respectively) or no ant colonies were found. As part of the field experiment, 50 artificial nest sites from each of the three groups were set in place in early spring 2020 and were then collected about two months later. Three of the nest sites (one from each group) were not included in the analysis, as during the experiment they had been pushed into the ground and their holes were blocked with soil.

**Figure 6:**
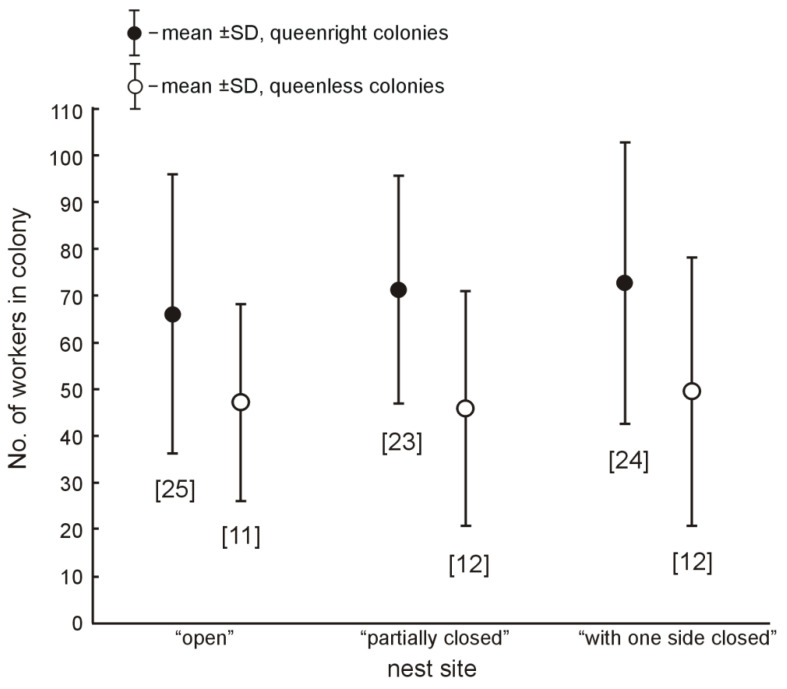
Number of workers of *Temnothorax crassispinus* queenright and queenless ant colonies found inside the ‘open’, ‘partially closed’, and ‘with one side closed’ artificial nest sites (see Figure 1 and Figure 5). The numbers of colonies are given in brackets.

On 27 May 2020, all of the 150 woodblocks were collected, but three of them (one from each group) had been pushed into the ground and their holes were blocked with soil. In 112 of the 147 remaining nest sites, signs of hole modifications were found. From the 112 nest sites with signs of a hole modification, ant colonies were present inside 107 cavities (35 queenless, workers: 5–109, mean: 47.5; and 72 queenright, workers: 8–149, mean: 70.0). Inside six more nest sites, 1–7 workers without a brood were found, and no ants were found in other nest sites. Contrary to the prediction, there was no difference in the proportion of inhabited nest sites from the three experimental groups (*χ^2^* = 0.12, *df* = 2, *p* = 0.94, analyzing number of the three types of nest sites: queenless colonies, queenright colonies, and sites without ant colonies; see Figure 5). The queenless colonies were smaller than the queenright colonies (*F* = 16.26, *df* = 1, *p* < 0.0001), but there were no differences in the number of workers in the colonies from the three types of nest sites (*F* = 0.36, *df* = 2, *p* = 0.70; see Figure 6).

27 of the 36 ‘open’ and 27 of the 35 ‘partially closed’ nest sites containing ant colonies had two entrances. For the queenright colonies inhabiting the ‘open’ or ‘partially closed’ nest sites (for such nest sites, the colonies were able to create two nest entrances), there was no difference in the colony size (i.e., number of workers) for the type of site (*F* = 0.47, *df* = 1, *p* = 0.49), nor for the sites with a different number of entrances (*F* = 0.71, *df* = 1, *p* = 0.40, analysis for the nest sites with one and two entrances).

The modified entrances to the nest sites were round or elliptical in shape, with areas ca. 0.8–3.8 mm^2^ (median: 1.5 mm^2^, N = 140). For the entrance modifications, or just blocking the hole, the ants used soil (in all 171 analyzed samples of the materials used for modification), grains of sand (103/171), and parts of plants (166/171) including pieces of leaves, stems, and seed husks, as well as pieces of bark and moss. Additionally, small parts of wood from the woodblocks were used in six cases. Typically, the structure of the material in the holes was strong, as though it had been cemented.

### 3.2. Experiment 2 (Field Experiment): During Nest Repairs, Do Ant Colonies Create Additional Entrances?

In the 39 nest sites with signs of hole modifications that were opened on 17 May, at the end of the field experiment, ant colonies were found in 31 sites (six queenless, 25 queenright, workers: 10–132, median: 65, mean: 64.1). Inside the next six nest sites, only one to three workers without a brood were present, and two sites contained no ants. In the nest sites where no signs of ant habitations were noticed on 17 May, no ants were found at the end of the experiment. 

For the 31 nest sites with ant colonies, four of the sites had one entrance (in all cases, the entrance was at the initial hole; the second hole, i.e., opened on 17 May, was completely blocked), 14 nest sites had two entrances (the initial one, and on the other side), and for the remaining 13 sites it was not possible to state definitely. In several cases, I observed that workers used both holes as entrances, when two were visible. The entrances to the nest sites were round or elliptical in shape, with areas ca. 0.8–3.1 mm^2^ (median 1.33, N = 37). For nine nest sites, in which both entrances were measured, there was no difference in the sizes of the entrances (the paired-sample Student *t* test, *t* = 0.59, *df* = 8, *p* = 0.57). Similar to the nest sites from Experiment 2, for the entrance modification or blocking the hole, the ants used soil (in 59 of the 61 analyzed samples of materials from the nest entrance modification), grains of sand (54/61), and parts of plants (61/61) including pieces of leaves, stems, seed husks, and pieces of bark. 

### 3.3. Experiment 3 (Laboratory Experiment): Do the Ant Colonies Prefer a Nest Sites with One Entrance vs. a Nest Site with Two Entrances?

After 48 h, 18 of the 34 colonies used during the experiment inhabited the artificial nest cavities: eight colonies inhabited a cavity with one entrance, while another eight inhabited a cavity with two entrances, and two of the colonies split into two available cavities. Six days after the beginning of the experiment, 27 of the 34 colonies inhabited the artificial nest cavities: 14 occupied a cavity with one entrance, 12 occupied a cavity with two entrances, and one colony remained split into two available cavities (the other colony, which had split after two days, finally chose a cavity with two entrances); the remaining seven colonies did not inhabit the artificial nest cavities. Thus, the ant colonies showed no preference for the nest sites with one entrance vs. the nest cavities with two entrances (*χ^2^* = 0.00, *df* = 1, *p* = 1.00, and *χ^2^* = 0.15, *df* = 1, *p* = 0.69 for the data after two and six days, respectively; analyses for the 16 and 26 colonies that chose one nest cavity). 

The ant colonies used grains of sand to reduce the size of all the entrances. The grains of sand used for the entrance size modifications were placed loosely. In each case, for the colonies that inhabited the nest sites with two entrances, the workers used both entrances. 

## 4. Discussion

For their nest sites, acorn ants may use acorns or cavities in twigs, but also empty streams, cavities under bark (e.g., [2,18]), and—if there is the lack of a good cavity—they are able to dig out a cavity in the soil [30]. During the field experiments, most of the artificial nest sites were inhabited by the ant colonies. This is in accordance with the findings of previous studies, that good places for nesting are limited resources for the acorn ants [13,15]. The colonies not only inhabited most of the artificial cavities, but also decreased the size of the initial holes. The cavity nesting ants typically inhabit acorns, galls, and small twigs, where the larvae of other insects have bored a cavity [4,19,29,32]; thus, such cavity usually has only one hole, which can be used by the ants as an entrance. During the field experiment, the ants often created two entrances to an artificial cavity; however, no data on the use of multiple entrances by the ant species are available. 

Typically, when looking for and collecting the ant colonies, twigs and acorns—the potential nest sites for such species—are collected and opened to check if ants are present inside. During such a procedure, it is not easy to check whether the cavity has only one entrance: the hole (potential entrance) is generally well-visible in acorns, but not in twigs. However, even for acorns, to find if a nest cavity has more than one entrance, it would be necessary to observe if the workers use more than one hole, as—for example—a visible hole could be blocked inside the acorn. During the field research, I occasionally observed acorns with (probably) two entrances, and I have found ant colonies inside such acorns. However, I have no data on whether both holes were used simultaneously as entrances. 

The sizes of the modified entrances to the artificial nest cavities, measured during the study, were similar to the findings of other studies for *Temnothorax* ants (e.g., *T. longispinosus* [14], *T. curvispinosus* [20], and *T. crassispinus* [30]). Under natural conditions, *Temnothorax* ants typically use soil for the entrance size reduction [20,30]. In a laboratory, they often decrease the entrances to an artificial nest cavity by using materials such as pieces of wood from natural nest site (acorns, twigs), if available, and readily use grains of sand [30]. Entrance modifications were found for *Temnothorax* ants inhabiting galls [19] as well as for a majority of the tropical arboreal ant species that dwell in tree cavities; for nest entrance reduction, different ant species can use a variety of different materials [16]. During the field experiments, the *T. crassispinus* ant colonies used soil and grains of sand, as well as parts of plants, and in almost all cases, all these types of materials were used. However, these were probably just the most frequent materials in the area. 

If the potential nest cavities had two large holes, the ant colonies would frequently prepare two entrances, and if two nest entrances were present, they were similar in size (see the results of Experiments 1 and 2). Further, during the laboratory experiment, contrary to the prediction, the colonies frequently inhabited the sites with two entrances and used both. No data were obtained to show if the nest sites with two entrances are better; however, many ant species use nests with single or multiple entrances, where the number of entrances can be diverse even inside the nests of one species (e.g., [38,39,40,41]). Using entrances from two opposite sites could have an influence on the foraging activity. For example, it was shown that for the red ant *Myrmica rubra*, the number of nest entrances influenced the collective foraging of the colony: multiple nest entrances reduced the foraging efficiency [42] but favored the simultaneous exploitation of several sources. It has been suggested that multiple entrances appear as a way for small- or medium-sized colonies to benefit from some of the advantages conferred by polydomy [38]—i.e., the simultaneous use of several nest sites by one colony—and *T. crassispinus* colony might use more than one nest (seasonal polydomy) [43]. It would be interesting to check if, for small colonies like those of *Temnothorax* ants, the effect of two entrances vs. one entrance on foraging is visible. Nevertheless, having a cavity with more than one entrance could be helpful if, for example, such a nest site is relocated and, after the relocating, the entrance situated on the bottom of the nest is blocked. It is easy to imagine such a situation in the case of acorns, which are typically inhabited by the ant colonies. This problem needs studying further, as many studies have shown that ants prefer smaller entrances (probably because they are easier to protect), while two entrances should be more difficult to protect than one.

As was stated above, during the laboratory experiment, the ants showed no preference and inhabited the cavities with one entrance vs. the cavities with two entrances in a similar proportion. However, even six days after the beginning of the experiment, seven of the 34 colonies used in the experiment were not inhabiting any of the available cavities—the colonies were still in the Petri dishes, outside the artificial cavities. This may suggest that the artificial nest sites used in the experiment were not easily accepted by the ants, but I have no data to provide an explanation for why this should occur. During numerous experiments in laboratories, various nest sites have been used [20,24,25,27,28,30]. However, the nest site design used in this case was different, as the nest site was prepared from several pieces of Plexiglas (see Figure 3). The pieces used were tightly joined, without a space between them. Thus, it was not the effect of using nest sites prepared from several pieces of Plexiglas. However, there could be another problem with such sites, e.g., the smell of the material that was used. Artificial nest sites differ from the natural ones. It was shown that, for example, conditions may vary even at different locations within the acorn nests [44,45], and the colony responds to different conditions, e.g., moving brood to the parts that stimulate brood development [45]. Nevertheless, as the nest sites with one and sites with two entrances were prepared using the same materials, it is clear that during the experiment, the colonies showed no preference for this variable. This is consistent with the findings from research using the ant *T. albipennis* [22] where, in general, the *Temnothorax* ants preferred nests with fewer entrances, but did not distinguish between the nest sites with one entrance and two entrances. 

During field experiments with acorn ants, artificial nest sites prepared from woodblocks are generally used, as the nest sites are limited resources for the acorn ants [13,15], and such cavities are willingly accepted by *Temnothorax* ants (e.g., [30,36,46] and the results of this study). For such experiments, the cavities are typically prepared by drilling a hole in a piece of wood, where such a hole is closed from one side and the other side is partially closed, as it has been shown that such ants prefer a nest site with a narrower entrance [33]. However, the results of this experiment showed that it is not necessary to prepare artificial cavities for the ants with an ‘ideal’ narrow entrance—as if the entrance is too large, the ants will still use such a cavity and modify the holes, if necessary. Furthermore, under natural conditions, such a narrow entrance can sometimes decrease in size after several days, as a result of soaking the plug used for the hole reduction (personal observation). However, using woodblocks that are drilled through (without blocking either side) could have an effect on the results of the experiment, as modifying the nest site (e.g., the entrance) requires work, time, and energy from the ants. Thus, for field experiments, preparing nesting cavities that are suitable for the ants could be an important factor affecting the experimental results. 

The results of the study showed that, though the ant colonies are small and the nest cavity is also small, acorn ants can create two entrances to a nest cavity. As the number of nest entrances could influence the ants’ behavior, more data are needed to determine whether the ant colonies simultaneously use more than one entrance under natural conditions, and if so, how this influences the behavior of the ants.

## Data Availability

The data presented in this study are available on request from the author.
